# Biochemical Assessments of Six Species of Edible Coastal Algae Collected from Tabuk Region in Saudi Arabia

**DOI:** 10.3390/molecules29030639

**Published:** 2024-01-30

**Authors:** Hala M. Bayomy, Eman S. Alamri

**Affiliations:** 1 Food Science and Nutrition Department, Science Faculty, University of Tabuk, Tabuk 71491, Saudi Arabia; ialamri@ut.edu.sa; 2 Food and Dairy Science and Technology Department, Faculty of Agriculture, Damanhour University, Damanhour 22516, Egypt

**Keywords:** edible algae, chemical composition, amino acids, oxidoreductive, Saudi Arabia

## Abstract

In the first study focusing on the Red Sea’s Tabuk coast, six edible species of the most common algae were collected to evaluate their approximate composition using AOAC methods, amino acids using ion-exchange chromatography, minerals using atomic absorption spectroscopy, phenolic compounds using the Folin–Ciocalteu method, and ferric-reducing antioxidant power. All the data were significantly (*p* < 0.05) different among all the studied species. The data indicated that the protein content ranged from 9.25% for *A. nodosum* to 20.06% for *H. musciformis. C. racemosa* had the highest lipid content of 7.57%. Phosphors varied from 68.2 mg/100 g for *A. nodosum* to 406 mg/100 g for *D. simplex*. The largest amounts of calcium (2458 mg/100 g) and iron (29.79 mg/100 g) were found in *C. racemosa*. The total essential amino acids ranged between 38.16 and 46.82% for *A. nodosum* and *D. simplex*, respectively. *F. vesiculosus* had the maximum content of phenolic compounds (11.06 mg GAE/g). *A. nodosum* had the highest antioxidant capacity (1.78 mg TE/g). The research concluded that algae are the main effort toward sustainable agriculture to meet the world’s food needs. that algae may be used to improve food naturally. To satisfy the criteria for sustainable food, which is one of the pillars of NEOM, numerous studies are required to investigate the natural products available in the Red Sea.

## 1. Introduction

In recent years, the Red Sea has attracted attention due to its biological diversity and the abundance of algae biomass [1]. The type and distribution of the algae in the Red Sea are contingent upon various factors, including temperature and salinity changes, the depth zone, and the season [2,3]. Gomez-Zavaglia et al. [3] classified algae into microalgae and macroalgae. It should be noted that not all scientists agree on the division of algae, but the perspective is continually changing. One of the criteria for the classification of macroalgae is their pigmentation [4], which allows us to identify several large groups: red algae *Rhodophyta* (more than 6000 species), brown algae *Phaeophyceae* (more than 2000 species), and green algae chlorophyta (more than 1200 species).

Green marine macroalgae possess highly diverse forms, such as single or multicellular. The plastids are stained green by chlorophyll a and b, linked to carotenoids and xanthophyll. *Caulerpa racemosa* and *Ulva lactuca* are edible green algae [5,6,7]. Brown algae possess a multicellular structure. Fucoxanthin is a highly prevalent pigment found in brown algae, outweighing other pigments such as chlorophyll-a, chlorophyll-c, β-carotene, and other xanthophylls [8]. Brown algae are a type of marine macroalgae; they are plant species found in the Red Sea and in coastal regions all over the world [9]. Some species, including *Ascophyllum nodosum* and *Fucus vesiculosus*, are eaten in Asian countries in traditional recipes such as sushi [10]. Red marine macroalgae are multicellular, and a few are single-cellular. Their color is due to the presence of pink plastids, in which the red pigment, phycoerythrin, is bound to several other pigments, including chlorophyll. *Digenea simplex* and *Hypnea musciformis* are red edible algae [11].

Scientists have noted that marine macroalgae can be considered a valuable food resource due to their antioxidant activity, which is beneficial for human health [12]. It prevents the formation of free radicals, which are responsible for oxidative stress and cause multiple diseases [13,14]. Several studies have confirmed the positive correlation between free radicals and cancer, circulatory diseases, aging, rheumatic arthritis, and nervous system diseases [7,15].

Several authors have found that algae contain a high level of vital nutrients, including proteins, amino acids, polyunsaturated fatty acids, and polysaccharides, in addition to dietary fiber, vitamins, and minerals [7,8,13]. Algae has long been considered a staple in the Asian diet, adding to its high nutritional value [7,8,13]. In the last decade, numerous species of sea algae have been shown to contain various types of antioxidant substances—for example, sulfated polysaccharides, catechins, sterols, proteins, phlorotanins, and carotenoid pigments such as fucoxanthin and astaxanthin [16]. In addition, the polyphenols that are available in algae show great potential and possess stronger antioxidant activity than terrestrial plants [7,17,18]. Some researchers have found a correlation between algae’s antioxidant activity and total phenolic compound levels [19]. The availability of such compounds in algae can protect the body from several illnesses and delay the aging process [20].

The chemical composition varies between algae species, which can be attributed to several factors, including salinity, temperature, location, light, seasonal period, and storage conditions [21,22]. Furthermore, algae are a great source of protein, which varies between 5% and 47% of the dry basic [7]. Recently, algae have been viewed as a cheap and innovative protein source with high nutritional quality [23].

The Red Sea is considered one of the most important regions of biological diversity on Earth and has a wide variety of algae species, but the nutritional benefits and chemical composition of the Red Sea algae are poorly understood. Therefore, the current study presents the first published data on the approximate composition, amino acids, minerals, phenolic compounds, and oxidoreductive compounds in common edible algae found on the Tabuk coast of Saudi Arabia. The investigation aims to identify the most prevalent edible algae on the Tabuk coast of the Red Sea and to estimate their nutritional composition for use in complementary studies in the near future.

## 2. Results

### 2.1. Proximate Chemical Composition

The calculated protein, lipid, ash, fiber, and carbohydrate percentages were derived from an ANOVA and are presented in Table 1 on a dry basis (db). Significant differences (*p* < 0.05) were found between all the species under study, which were composed of a wide variety of nutrients. The red algae *H. musciformis* had the highest protein content (20.06%, db), and the lowest value (9.25%, db) was found in the brown algae *A. nodosum*. The lipid content in the present study ranged from 0.93 to 7.57% for *D. simplex* and *C. racemosa*, respectively. The ash and crude fiber contents were higher among the algae species under study. The ash content varied from 13.64% in *C. racemosa* to 29.38% for *A. nodosum*, while the crude fiber varied from 11.75 to 34.81% for *U. lactuca* and *H. musciformis*, respectively. *H. musciformis* had the lowest carbohydrate content (23.56%), whereas *U. lactuca* had the highest content (54.52%).

### 2.2. Minerals

The mineral content of the six algae under study is given in Table 2. The values illustrated significant differences (*p* < 0.05) among all species. The highest calcium value (2458 mg/100 g) was found in the *C. racemosa* species, whereas the lowest value (476 mg/100 g) was found in *D. simplex.* On the other hand, the phosphorus content ranged between 68.29 and 747 mg/100 g for *U. lactuca* and *C. racemosa*, respectively. The iron content varied from 16.85 to 29.79 mg/100 g for *A. nodosum* and *C. racemosa*, respectively. The highest potassium content (7496 mg/100 g) was found in *D. simplex*, while the lowest content (477 mg/100 g) was found in *H. musciformis*. The sodium content ranged from 406 to 6156 mg/100 g for *H. musciformis* and *U. lactuca*, respectively.

**Figure 1:**
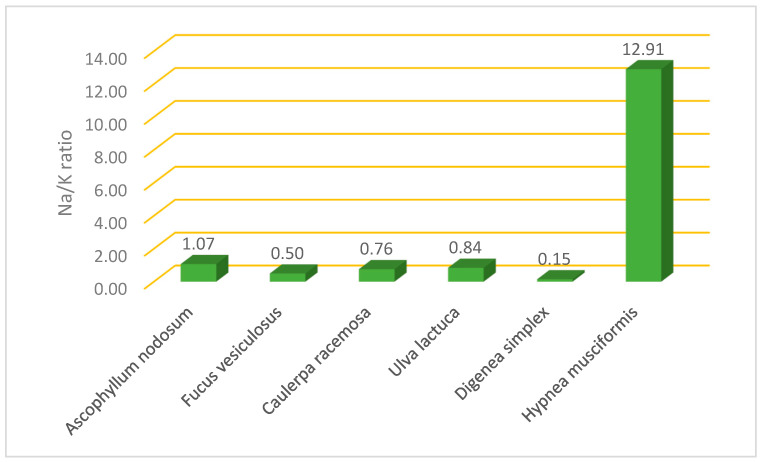
Na/K ratio in selected edible algae collected from Tabuk coast.

As shown in Figure 1, *D. simplex* had the lowest Na/K ratio (0.15), while *H. musciformis* presented the highest Na/K ratio (12.90).

### 2.3. Total Phenolic Content and Oxidoreductive Compounds

**Figure 2:**
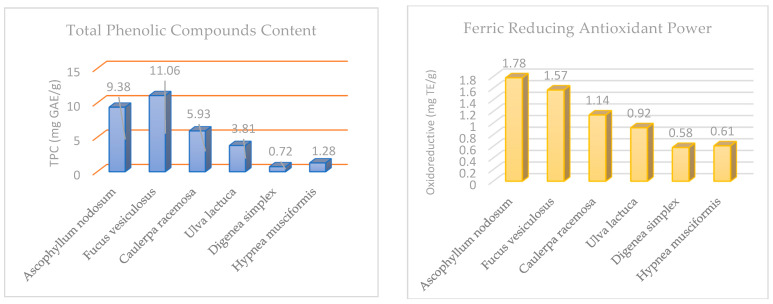
Total phenolic compounds (mg GAE/g) and ferric-reducing antioxidant power (mg TE/g) in selected edible algae.

Figure 2 shows the total phenolic content and oxidoreductive compounds of the six algae species taken from the Tabuk coast. The one-way ANOVA analysis showed that the oxidoreductive compounds differed significantly among the algae species under study. The brown species *F. vesiculosus* (11.06 mg GAE/g) and *A. nodosum* (9.38 mg GAE/g) had the highest levels of total phenolic compounds, followed by green algae, then the red algae *D. simplex* (0.72 mg GAE/g). *H. musciformis* (1.28 mg GAE/g) had the lowest level of total phenolic compounds. Among the tested algae, brown algae showed the highest reducing power (1.78 and 1.57 mg TE/g) for *A. nodosum* and *F. vesiculosus*, respectively, followed by green algae, and the lowest reducing power was observed in red algae *D. simplex* (0.58 mg TE/g) and *H. musciformis* (0.61 mg TE/g).

### 2.4. Amino Acids

The amino acid composition (g/100 g protein) for the selected edible algae is shown in Table 3. The total essential amino acids ranged from 38.16 to 46.82 g/100 g protein for *A. nodosum* (which had the highest level of non-essential amino acids, 61.64 g/100 g protein) and *H. musciformis* (which had the lowest level of non-essential amino acids, 52.98 g/100 g protein), respectively. The calculated EAA/total AA ratio varied from 38.24% in *A. nodosum* to 46.91% in *H. musciformis*.

## 3. Discussion

Ścieszka and Klewicka [24] note that algae are found throughout the world, covering two-thirds of all water bodies. Algae have been employed in a variety of food products due to their high concentrations of prebiotic and bioactive components. Agar, alginate, and carrageenan have been developed as a result of their gelling, thickening, and stabilizing qualities [7]. Furthermore, algae are utilized in food products as a dietary supplement and as an ingredient in functional foods, as well as to produce fermented foods. In addition, algae are used to improve the quality of meat products such as pasties, steaks, frankfurters, and sausages, as well as fish, fish products, and oils. Algae are also used to fortify cereal-based foods, including pasta, wheat, and bread. This was confirmed in [25], which summarized that algae is considered a real gold mine for many bioactive compounds, including protein, which is present in large proportions (about 30% to 55–60%), and therefore, it can be used to treat malnutrition, as more than 821 million people are malnourished globally due to inadequate protein-rich diets, resulting in increased demand for total dietary protein [25].

Babich et al. pointed out that the protein content varies greatly between different algae groups. Among the macroalgae, red and green algae (e.g., *P. vulgaris* and *U. lactuca*) often contain high levels of protein, in contrast to the low levels in most brown algae [26]. Fleurence [22] found that the protein content of most brown algae (*A. nodosum* and *F. vesiculosus*) used in industry is less than 15%, db. The protein content in certain green algae species, such as those in the genus *Ulva*, can range from 10% to 26%, db. However, several investigated red algae have been found to have higher protein levels for *Porphyra tenera* (47%, db) and *Palmaria palmata* (19.94%, db), as illustrated in [27,28]. The findings of the chemical composition analysis clearly show that the macronutrient content of algae varies. In our study, the highest content of protein and lipids was detected in the green algae *C. racemosa*. The highest content of protein and fiber, with a low content of lipids and carbohydrates, was found in *H. musciformis*. This indicates their great potential for use in the diet.

Our results are in line with those reported in [22,29,30]. According to [30], the proximate compositions of *C. racemosa* discovered on Martin Island were found as follows: protein, 19.72%; crude lipids, 7.65%; carbohydrates, 48.97%; fiber, 11.51%; and ash, 12.15. Moreover, in Lorenzo et al. [31], the protein, lipid, and ash content were recorded to be 8.70, 3.62, and 30.89%, db, respectively, for brown algae *A. nodosum* from the Spanish coast. Meanwhile, *F. vesiculosus* contained 12.99, 3.75, and 20.7% db protein, lipid, and ash content, respectively. The protein content of algae varies across the year, according to [2,32], with the highest levels in the winter and early spring and the lowest levels in the summer and early autumn. Our findings are in agreement with [33], who concluded that the chemical composition varies according to the species, geographical area, season, and estimation process. The chemical composition showed the high ash content of algae, indicating their high content of minerals. It was also found that all algae species in the current study contained many times more calcium than milk [26], indicating that they may be an excellent source of calcium for osteoporosis prevention and treatment, for developing children, and for pre- and postmenopausal women. The Ca content in all species under study was higher than that reported in [31] for *F. vesiculosus* (1160.27 mg/100 g). The analysis of the iron content indicated that the algae had greater iron content than many well-known dietary sources of iron, such as leafy green vegetables, legumes, nuts, and common cereals, which all contain between 2 and 4 mg/100 g [34]. One strategy to prevent iron deficiency, one of the most prevalent nutritional deficits around the world, could be to use these algae as a natural food source. The present results are in contrast to those of [34], which found that *A. nodosum* had 10 mg Fe/100 g, which was less than our result (16.85 mg/100 g), but *F. vesiculosus* and *U. lactuca* had 29 and 180 mg Fe/100 g, respectively, which were greater than our findings. All species of algae considered in the present study are good sources of potassium, especially *D. simplex* (7496 mg K/100 g, db), *F. vesiculosus* (3758 mg/100 g, db), and *A. nodosum* (3643 mg/100 g, db). The most prevalent element in seaweeds was K (3781.35–9316.28 mg/100 g db), followed by Na (1836.82–4575.71 mg/100 g db) and Ca (984.73–1160.27 mg/100 g db), according to [31]. Similar conclusions were reached in [35,36].

A low Na/K ratio is beneficial for human health as it reduces the risk of high blood pressure as well as cardiovascular disease [37]. The lowest Na/K ratio was found in *D. simplex*, so this species can be used as a flavorful alternative to table salt (NaCl) for those with hypertension and cardiovascular diseases. The same trend regarding the polyphenol content was found among all six species when studying the oxidoreductive compounds via the ferric-reducing antioxidant power assay.

**Figure 3:**
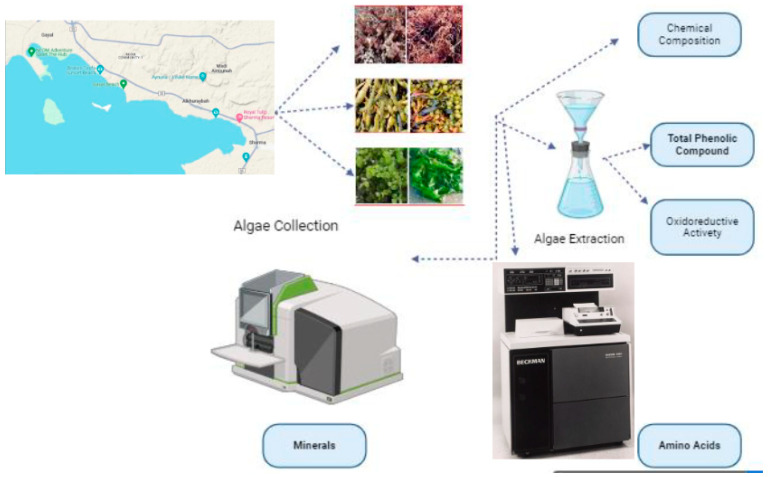
Flowchart to summarize materials and methods used.

Brown algae are the richest in polyphenols and show the greatest ability to scavenge free radicals (Figure 3), compared to the two other families of marine algae (green algae and red algae). This result is correlated with the findings of [38,39]. Phlorotannins represent the major phenolic compounds in marine brown algae [40]. It is known that phlorotannins have a variety of biological properties, such as the suppression of antiplasmin, heavy metal detoxification, antimicrobial activity, UV protection, and chemoprevention against vascular risk factors [17,41]. The authors of [42,43] demonstrated that the antioxidant activity was correlated with the total phenolics. Meanwhile, in [44], it was found that the oxidoreductive qualities of marine algae may arise from several bioactive compounds, including polyunsaturated fatty acids, especially omega 3, as well as pigments such as chlorophylls and carotenoids, vitamins, vitamin precursors, sulfated polysaccharides, and phenolic compounds, which are believed to be the most active elements responsible for marine algae’s antioxidant functions. Therefore, marine algae are a good source of both water- and fat-soluble antioxidants [7,45]. These aspects indicate that the naturally occurring antioxidant substances present in edible algae can both shield food items from oxidative deterioration and prevent and/or treat illnesses brought about by free radicals. As a result, it is important to evaluate how the most common cooking techniques (boiled, steamed, and pancake) affect the levels of carotenoids and chlorophylls, total phenolic compounds, and antioxidant capacity. This represents an important direction in our future research. The highest levels of non-EAA were recorded for aspartic acid, glutamic acid, and alanine in every algae species under study, in line with the results found in [31]. According to [46], the glutamic and aspartic acid contents are influenced by the specific flavor and taste of seaweed. The green alga *U. lactuca* from Norway [47] had an EAA% of 40.30–40.79, which is closely related to our study (39.67%).

The calculated EAA/total AA ratio for the present species was similar to that found in [48] for different brown and red Spanish edible seaweeds. This ratio reached 42.72%, 40.82%, and 36.87% for *Undaria pinnatifida*, *Halomonas elongata*, and *Porphyra umbilicalis*, respectively. The EAA/total AA ratio for the studied species, however, was lower than the figures provided in [35], who noted ratios over 55% in *Gracilaria changii*. Leucine, threonine, and lysine were the most abundant essential amino acids found in the studied algae, and our findings are in line with those recorded by Lorenzo et al. [31] for *F. vesiculosus* and *A. nodosum.* However, they are in contrast to those of [35], who considered arginine to be the most abundant EAA in *G. changii*. In all of the algae species analyzed, high-quality protein was found, suggesting that they could be used as a supplement for human nutrition. Most algae tend to provide adequate amounts of total essential amino acids within the required limits for food because of their high concentrations of essential amino acids, particularly lysine, which is a limited amino acid in many foods.

## 4. Materials and Methods

Materials and methods can be summarized in the following flowchart (Figure 3).

### 4.1. Materials

All employed chemicals, reagents, and solvents were of the highest purity and were purchased from the Sigma-Aldrich Company (St. Louis, MI, USA).

#### Sampling Description

**Figure 4:**
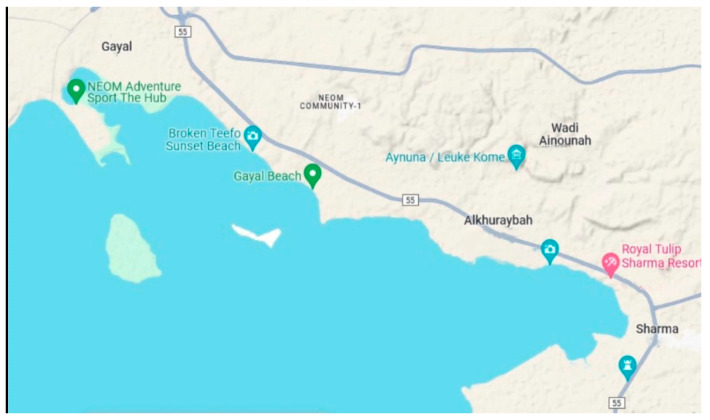
Selected coastal areas in the region of Tabuk (source: https://www.google.com/maps/@28.0766633,35.0502921,12z/data=!5m1!1e4?hl=en accessed on 22 January 2024).

**Figure 5:**
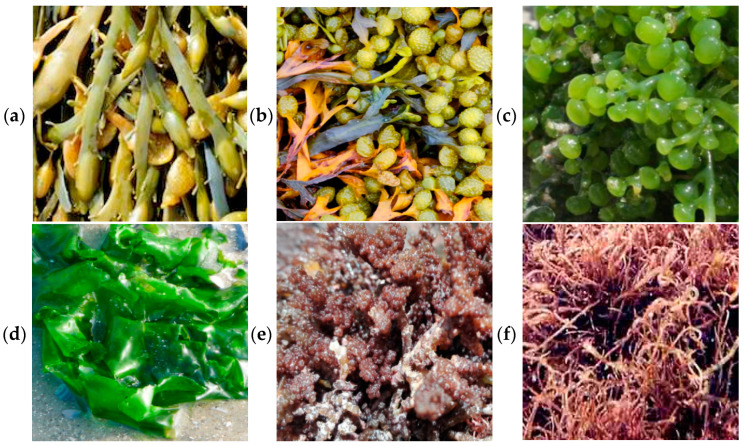
(**a**) *Ascophyllum nodosum*, (**b**) *Fucus vesiculosu*, (**c**) *Caulerpa racemosa*, (**d**) *Ulva lactuca*, (**e**) *Digenea simplex*, and (**f**) *Hypnea musciformis*.

Algae samples were collected during September 2020 from three selected coastal zones in the region of Tabuk, including Sharma (27°55′48.4320″ N and 35°16′38.3808″ E), Alkhuraybah (28°03′24.0″ N 35°09′50.9″ E), and Gayal (28°07′33.5″ N 35°01′40.1″ E), as marked in Figure 4. According to Ansari and Ghanem [2], summer is the best season for the collection of algae samples, reflecting the diversity and density of Red Sea algae. Six species of edible algae were selected, including *Ascophyllum nodosum* and *Fucus vesiculosus* as brown algae, *Caulerpa racemosa* and *Ulva lactuca* as green algae, and *Digenea simplex* and *Hypnea musciformis* as red algae (Figure 5), and we collected approximately 3 kg of each species from each zone. All the species were identified with the assistance of the employees of the Oceanography Department at Alexandria University. To eliminate any associated contaminants, water was used to wash the obtained samples; then, they were rinsed with sterile water and dried in a shady environment at ambient temperature to prevent photolysis and thermal degradation. Then, they were minced well; the minced samples were preserved in airtight glass jars under freezing and then brought to the laboratory in iced conditions.

### 4.2. Methods

Proximate Chemical Composition: The proximate chemical composition of the selected algae was analyzed using the methodology of AOAC No. 930.15 for moisture, AOAC 984.13 for crude protein, AOAC 2003.05 for lipids, AOAC 978.10 for crude fiber, and AOAC 942.05 for ash. Results are presented as a percentage on a dry basis. The carbohydrate content (nitrogen-free extract) was computed by subtracting the total percentage of crude protein, fat, crude fiber, and ash content from one hundred [49].

Minerals: The concentrations of Fe, Ca, P, Na, and K were determined after transferring white to gray ash using treatment with 6N hydrochloric acid (HCl), followed by injection using atomic absorption spectroscopy (AAS), in line with the method explained in [50].

Amino Acids: First, 5 g of the sample and 2.5 mL of 6N hydrochloric acid were poured into a hermetically sealed hydrolysis tube. The assembly was then brought to a temperature of 110 °C. After 72 h, the hydrolysis was complete, and we determined the amino acids using ion-exchange chromatography (Beckman 7300 High-Performance Amino Acid Analyzer, Inc., Palo Alto, CA, USA). The alkaline hydrolysis of a sample was used to determine the tryptophan content [25].

#### 4.2.1. Extraction Process for Algae Samples

The method recommended by Hemalatha et al. [51] was adopted to obtain the extracts, with some modifications. First, 2 g of the freeze-dried powder of different macroalgae was soaked in 40 mL of methanol for 24 h at ambient temperature. Twice more, extraction was performed, with the extracts filtered using Whatmann No. 1 filter paper (Sigma-Aldrich, St. Louis, MO, USA) each time, and the supernatants were kept at 4 °C to perform the analysis of total phenolics and oxidoreductive activity.

#### 4.2.2. Total Phenolic Compounds

The total phenolic content in the selected algae was estimated using the Folin–Ciocalteu method, as explained by Dang et al. [52]. A spectrophotometer was used to detect the absorbance at 725 nm in comparison to a solvent blank. The total phenolic content was estimated using a calibration curve generated with gallic acid concentrations ranging from 4 M to 0.5 mM and represented as (mg GAE/g).

#### 4.2.3. Determination of Ferric Reducing Antioxidant Power

The ferric-reducing antioxidant power (FRAP) assay was used. This procedure is based on the reduction of a ferric-tripyridyl triazine complex to its ferrous-colored state after incubation at 37 °C for 10 min in the presence of antioxidants. The absorbance of the reaction mixture at 593 nm was determined using a spectrophotometer. Calibration was performed using a series of concentrations between 200 and 1000 μM of FeSO_4_ 7H_2_O to plot the standard curve. FRAP was expressed as mg TE/g [53].

#### 4.2.4. Statistical Analysis

The obtained results were recorded as the average of three replicates ± SD (standard deviation) except the data of amino acid, with the exception of amino acids, using Duncan’s multiple range test with a one-way analysis of variance (ANOVA) at the significance level of *p* < 0.05. SPSS (version 21.0) was used.

## 5. Conclusions

Among all types of edible algae collected from Tabuk on the Red Sea coast, a high content of protein (18.39%), lipids (7.57%), ash (13.64%), crude fiber (13.83%), calcium (2458 mg/100 g), phosphorus (747 mg/100 g), and iron (29.79 mg/100 g) was observed in green algae *C. racemosa*, while a high content of protein (20.06%) and fiber (34.81%) with a low content of lipids (1.83%) and carbohydrates (23.56%) was found in *H. musciformis*. These possess great potential for use in the diet. All species of algae considered in the present study are good sources of potassium, especially *D. simplex*, which had the lowest Na/K ratio, while *H. musciformis* presented the highest Na/K ratio. Therefore, *D. simplex* can be used as a flavorful alternative to table salt (NaCl) for those with hypertension and cardiovascular diseases. The brown species *F. vesiculosus* and *A. nodosum* had the highest levels of total phenolic compounds and oxidoreductive activity, as determined using the ferric-reducing antioxidant power assay, followed by green algae and red algae. The natural antioxidant compounds found in edible algae can protect food products from oxidative degradation, as well as prevent and/or treat diseases caused by free radicals. Leucine, threonine, and lysine were the most abundant essential amino acids found in the algae under study. In all the algae species analyzed, high-quality protein was also found, suggesting that they could be used as a supplement in human nutrition. Moreover, most algae tend to provide adequate amounts of total essential amino acids within the required limits for food because of their high concentrations of essential amino acids, which ranged between 38.16 and 46.82% for *A. nodosum* and *D. simplex*, respectively. *F. vesiculosus* had the maximum content of phenolic compounds (11.06 mg GAE/g). *A. nodosum* had the highest antioxidant capacity (1.78 mg TE/g). The study concludes that the nutrient content of algae varies and that the Red Sea algae can be used as a natural food supplement that is rich in many nutrients. However, there is a need for additional studies to explore the natural products of the Red Sea and their potential to fulfill the requirements of healthy food, which is one of the pillars of the NEOM national project.

## Figures and Tables

**Table 1:** Proximate chemical composition (% *w*/*w*, db) of dried edible algae collected from Tabuk coast.

Type	Species	Protein	Lipid	Ash	Crude Fiber	Carbohydrate
Brown algae	*Ascophyllum nodosum*	9.25 ± 0.62 ^d^	3.96 ± 0.07 ^c^	29.38 ± 0.44 ^a^	24.26 ± 0.53 ^bc^	33.15 ^cd^
	*Fucus vesiculosus*	13.71 ± 0.17 ^cd^	3.74 ± 0.01 ^c^	21.04 ± 0.31 ^b^	19.61 ± 0.17 ^c^	41.9 ^bc^
Green algae	*Caulerpa racemosa*	18.39 ± 0.72 ^ab^	7.57 ± 0.38 ^a^	13.64 ± 0.16 ^c^	13.82 ± 0.08 ^d^	46.58 ^ab^
	*Ulva lactuca*	14.62 ± 0.47 ^cd^	5.29 ± 0.13 ^b^	13.82 ± 0.06 ^c^	11.75 ± 0.14 ^d^	54.52 ^a^
Red algae	*Digenea simplex*	15.17 ± 0.24 ^bc^	0.93 ± 0.03 ^d^	23.62 ± 0.77 ^ab^	32.42 ± 1.21 ^ab^	27.86 ^cd^
	*Hypnea musciformis*	20.06 ± 0.53 ^a^	1.83 ± 0.08 ^d^	19.74 ± 0.52 ^b^	34.81 ± 1.05 ^a^	23.56 ^d^

Mean ± standard deviation. Different letters in each column indicate statistically significant differences (*p* < 0.05).

**Table 2:** Mineral content in mg/100 g of dried edible algae collected from Tabuk coast.

Type	Species	Ca	P	Fe	K	Na
Brown algae	*Ascophyllum nodosum*	1026 ± 31.3 ^b^	185 ± 28.5 ^c^	16.85 ± 0.16 ^c^	3643 ± 84.8 ^b^	3895 ± 204 ^b^
	*Fucus vesiculosus*	1049 ± 27.1 ^b^	208 ± 9.3 ^c^	20.66 ± 2.04 ^b^	3758 ± 61.8 ^b^	1869 ± 64.7 ^d^
Green algae	*Caulerpa racemosa*	2458 ± 39.7 ^a^	747 ± 13.6 ^a^	29.79 ± 1.94 ^a^	2873 ± 68.3 ^c^	2188 ± 39.3 ^c^
	*Ulva lactuca*	2393 ± 3.42 ^a^	68.29 ± 1.6 ^d^	25.63 ± 0.13 ^a^	481 ± 23.7 ^d^	406 ± 14.8 ^f^
Red algae	*Digenea simplex*	476 ± 22.4 ^c^	406 ± 17.2 ^b^	18.03 ± 0.79 ^bc^	7496 ± 142.7 ^a^	1098 ± 15.7 ^e^
	*Hypnea musciformis*	647 ± 8.2 ^c^	371 ± 24.5 ^b^	20.3 ± 0.86 ^b^	477 ± 54.8 ^d^	6156 ± 173.6 ^a^

Mean ± standard deviation. Different letters in each column indicate statistically significant differences (*p* < 0.05).

**Table 3:** Amino acid profile (g/100 g protein, db) for selected algae under study.

Amino Acids	*Ascophyllum nodosum*	*Fucus* *vesiculosus*	*Caulerpa* *racemosa*	*Ulva* *lactuca*	*Digenea simplex*	*Hypnea* *musciformis*	Standard Error
Aspartic acid	9.35 ^d^	12.29 ^b^	10.37 ^c^	13.06 ^a^	10.49 ^c^	12.02 ^b^	0.646
Serine	5.11 ^c^	6.74 ^b^	5.35 ^c^	7.72 ^a^	5.36 ^c^	6.32 ^b^	0.255
Glutamic acid	18.63 ^a^	14.41 ^b^	12.29 ^c^	11.5 ^c^	10.33 ^d^	12.19 ^c^	0.803
Proline	4.34 ^d^	5.83 ^a^	4.56 ^c^	3.37 ^f^	4.7 ^b^	4.18 ^e^	0.051
Glycine	6.53 ^a^	4.75 ^d^	6.34 ^b^	5.83 ^c^	5.76 ^c^	4.31 ^e^	0.171
Alanine	8.12 ^a^	7.19 ^b^	7.96 ^a^	6.98 ^b^	5.76 ^c^	5.04 ^d^	0.222
Arginine	4.55 ^d^	5.07 ^c^	6.83 ^b^	7.31 ^a^	4.19 ^e^	5.41 ^c^	0.341
Cysteine	0.24 ^e^	1.53 ^a^	0.39 ^d^	0.87 ^c^	1.04 ^b^	1.05 ^b^	0.045
Tyrosine	4.77 ^b^	3.39 ^e^	3.75 ^c^	3.52 ^d^	8.62 ^a^	2.46 ^f^	0.098
Total Non-EAA	61.64	61.2	57.84	60.16	56.25	52.98	0.327
Methionine	2.61 a	1.59 ^c^	1.6 ^c^	1.56 ^c^	0.79 ^d^	2.14 ^b^	0.130
Isoleucine	4.21 ^c^	3.71 ^d^	6.17 ^a^	3.86 ^d^	4.7 ^b^	6.18 ^a^	0.029
Leucine	5.84 ^d^	6.79 ^b^	7.04 ^a^	5.92 ^d^	6.42 ^c^	7.64 ^a^	0.140
Phenylalanine	3.70 ^e^	3.95 ^d^	4.86 ^b^	4.94 ^b^	6.09 ^a^	4.5 ^c^	0.093
Histidine	4.37 ^a^	3.92 ^b^	2.48 ^d^	2.55 ^d^	3.11 ^c^	4.31 ^a^	0.080
Valine	5.84 ^a^	4.76 ^e^	4.69 ^f^	5.42 ^b^	5.03 ^d^	5.23 ^c^	0.011
Lysine	3.88 ^f^	5.84 ^c^	6.41 ^b^	4.83 ^e^	9.21 ^a^	5.36 ^d^	0.037
Tryptophan	1.76 ^e^	2.31 ^c^	2.07 ^d^	1.76 ^e^	2.52 ^b^	2.99 ^a^	0.046
Threonine	5.95 ^d^	5.47 ^f^	6.73 ^c^	8.83 ^a^	5.63 ^e^	8.47 ^b^	0.103
Total EAA	38.16	38.34	42.05	39.67	43.5	46.82	0.720
EAA/Total AA ratio	38.24	38.52	42.10	39.74	43.61	46.91	

Values are means of triplicates. Different letters in each row indicate statistically significant differences (*p* < 0.05).

## Data Availability

Data are contained within the article.
